# Dawn of a new era of diabeto‐oncology

**DOI:** 10.1111/jdi.13204

**Published:** 2020-01-29

**Authors:** Hiroshi Noto

**Affiliations:** ^1^ Endocrinology Department St. Luke's International Hospital Tokyo Japan

## Abstract

Pathways of the mutual relationship between diabetes and cancer.

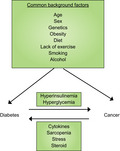

Diabetes mellitus and cancer have several risk factors in common (Figure 1). Besides these shared factors, they might directly trigger each other. Compelling evidence has been accumulated over the past decades that shows the increased risk of cancer in relation to diabetes in Western and Asian countries^1^. It is postulated that hyperglycemia and compensatory hyperinsulinemia in type 2 diabetes mellitus patients are the prevailing mechanisms through which diabetes directly raises cancer risk (Figure 1).

**Figure 1:**
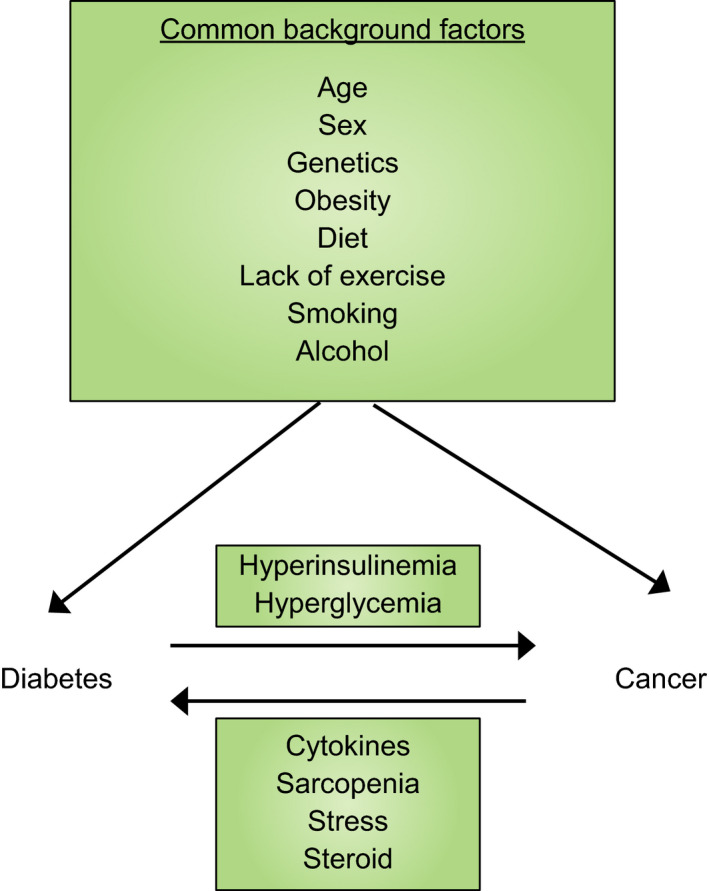
Pathways of the mutual relationship between diabetes and cancer. Several common factors are associated with a concomitant increase in the risks of diabetes and cancer. Additionally, the risk of cancer in diabetes is elevated through hyperinsulinemia and hyperglycemia. In contrast, patients with cancer are more susceptible to diabetes secondary to tumor‐related cytokines, sarcopenia, stress and steroid use.

However, the mechanism has not been fully elucidated and epidemiological studies (“real world data”) need to be interpreted with caution, because there might remain room for confounding bias, detection bias, channeling bias and reverse causality, which may could to overestimation of the magnitude of the risk. In fact, only a minority of links between diabetes and cancer risk has robust supporting evidence without bias. A recent retrospective longitudinal observational study showed that there was no association between glycemic level and the development of subsequent malignancies^2^, which failed to prove a dose–response effect of hyperglycemia and to negate reverse causality. Of interest, however, another analysis using the identical database showed that visit‐to‐visit glycated hemoglobin variability was dose‐dependently associated with cancer development in patients with diabetes^3^, pointing to a dynamic effect of glucose on oncogenesis. In accordance with this hypothesis, the risk of cancer death reportedly elevates in parallel with postprandial hyperglycemia^4^.

Maintaining good management of diabetes to correct hyperglycemia/hyperinsulinemia would presumably obviate the excess risk of cancer in diabetes. However, the spin‐off analyses of pivotal randomized controlled trials on cardiovascular risk suggested a non‐significant association between glycemic control and cancer risk, and high‐quality evidence is scarce to date^5^.

At present, evidence on the risk of cancer in association with any diabetes treatment is limited to substantiate the causal relationship between antidiabetic agents and cancer because of their inadequate adjustment for confounding factors, or not accounting for the degree of exposure of medications. Exceptionally, more attention has been focused on a potential anti‐oncogenic property of metformin. It is an insulin sensitizer, and lowers the levels of insulin and glucose in the blood, which would result in a lower cancer risk. In addition, metformin presumably suppresses oncogenes, such as mammalian target of rapamycin, and several other mechanisms are also postulated. A meta‐analysis of observational studies and randomized controlled trials showed that metformin use was associated with a 33% lower risk of cancer incidence and mortality, although this magnitude might have been overestimated due to channeling bias^6^. Interventional trials are ongoing to corroborate the direct anti‐tumor property of metformin. A pilot small randomized controlled trial suggested that small‐dose metformin exerted a favorable effect on an endoscopic marker of colon cancer in non‐diabetic people, and a phase II trial showed a significant benefit in 1‐year progression‐free survival with the adjuvant use of metformin in patients on chemotherapy for advanced or metastatic non‐squamous non‐small cell lung cancer^7^. Further studies are eagerly awaited, and it would be prudent at least for now to instruct diabetes patients to follow healthy lifestyles and to encourage them to have evidence‐based cancer screening.

Conversely, cancer is known to potentially increase the risk of diabetes. The favored mechanisms include the secretion of some cytokines by tumor cells, an increase in insulin resistance through sarcopenia, marked physical/ psychological stress and the use of steroids (Figure 1). A recent large long‐term cohort study showed that participants who developed cancer had a significant increase in the subsequent risk of diabetes, calling for routine diabetes screening in these patients^8^. In fact, it is reported that concomitant diabetes is one of the major determinants of quality of life in cancer, and it is related to increased mortality in patients with cancer. However, optimal management of diabetes in such patients is unknown, and further research is urgently required to fully elucidate the specific mechanisms and the natural history.

The die is cast. It is high time for diabetologists and oncologists to clinically collaborate with each other in this new field of “diabeto‐oncology”.

## Disclosure

The author has received lecture fees from Eli Lilly and MSD.
